# Examining spatial heterogeneity in built environment and climate factors affecting motorcycle crashes

**DOI:** 10.1371/journal.pone.0346916

**Published:** 2026-04-27

**Authors:** Yaqiu Li, Junyi Zhang, Haoran Li, Lon Virakvichetra

**Affiliations:** 1 School of Transportation, Southeast University, Nanjing, China; 2 Graduate School of Advanced Science and Engineering, Hiroshima University, Higashi Hiroshima, Japan; 3 Tsinghua University Suzhou Automotive Research Institute, Suzhou, China; 4 School of Automotive and Transportation Engineering, Wuhan University of Science and Technology, Wuhan, China; Sri Lanka Institute of Information Technology, SRI LANKA

## Abstract

Motorcycle crashes are a major contributor to road traffic fatalities in Cambodia, where motorcycles represent the dominant mode of transportation. Given the spatial dependence and heterogeneity inherent in crash data, this study examines spatial associations between built environment characteristics, climatic factors, and motorcycle crash frequency across 197 districts in Cambodia in 2019. Global Moran’s Index was used to assess spatial autocorrelation in crash frequency and explanatory variables. After evaluating the distributional properties of crash counts and multicollinearity among predictors, several regression models were estimated and compared, including Ordinary Least Squares regression (OLS), Poisson regression (PR), Negative Binomial regression (NBR), and Geographically Weighted Negative Binomial Regression (GWNBR). The results indicate that the GWNBR model outperforms global models by more effectively capturing spatial heterogeneity in the relationships between environmental factors and motorcycle crash frequency. Several variables exhibit relatively consistent spatial association patterns across districts: road length, road density, residential land use proportion, and precipitation are positively associated with motorcycle crash frequency in many locations, whereas population density, intersection density, and the number of annual rainy days are predominantly negatively associated. By revealing spatially varying association patterns in motorcycle crashes, this study provides evidence to support geographically differentiated approaches to motorcycle safety analysis and planning in Cambodia and other low- and middle-income countries.

## 1. Introduction

**Fig 1:**
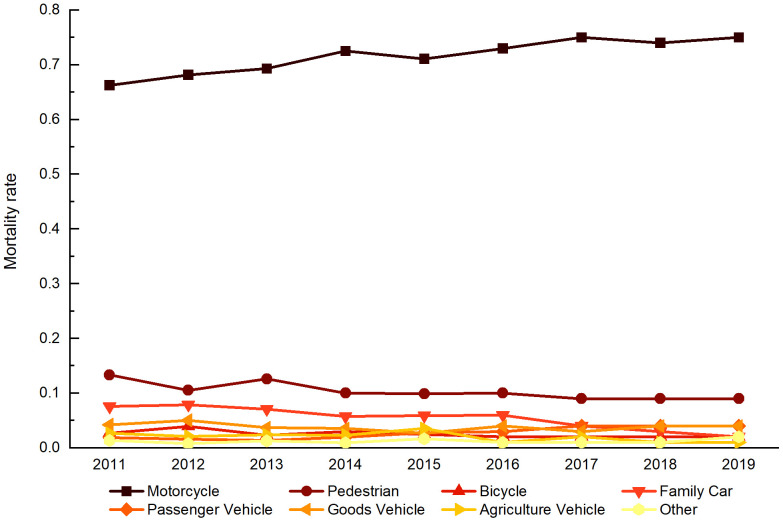
Annual mortality rate by transportation mode in Cambodia.

According to the World Health Organization [1], road traffic fatalities and injuries continue to be a major health and development concern worldwide, with pedestrians, motorcyclists, and cyclists accounting for over half of these deaths. These vulnerable road users’ susceptibility to traffic incidents is markedly higher relative to automobile drivers, attributable to their increased exposure to environmental risk factors. WHO [1] also demonstrates that Southeast Asia Region accounts for 28% of global road traffic deaths, with a staggering 92% of these fatalities occurring in countries with low to middle income levels. Within the Southeast Asian region, Cambodia exhibits the highest incidence of motorcycle-related fatalities, surpassing neighboring nations such as Thailand, Malaysia, and Myanmar [2]. Fig 1 indicates that more than 65% of road traffic related fatalities in Cambodia involve motorcyclists. Motorcycles dominate as the primary mode of transportation in Cambodia, with annual motorcycle registrations consistently accounting for over 75% of total registrations between 2011 and 2019 [3]. Additionally, there has also been a substantial increase of 66.67% in motorcycle registrations during the same period [3], which highlights the importance of conducting research on motorcycle crashes and implement measures to mitigate them.

The Cambodia National Road Safety Committee Report provides a comprehensive analysis of road crash fatalities across the twenty-five provinces of Cambodia for the years 2016 and 2017 [4]. This report highlights a disparate spatial distribution of fatalities, with Phnom Penh experiencing the highest number, followed by the Kandal and Kompong Thom provinces. Notably, while Phnom Penh observed a 9% decline in road fatalities from 2016 to 2017, the Kandal province experienced a substantial 45% increase during the same period. Concurrently, Kampong Thom province also witnessed a 6% escalation in road crash fatalities. This data underscores the geographical variability in road safety trends across Cambodia’s provinces.

**Fig 2:**
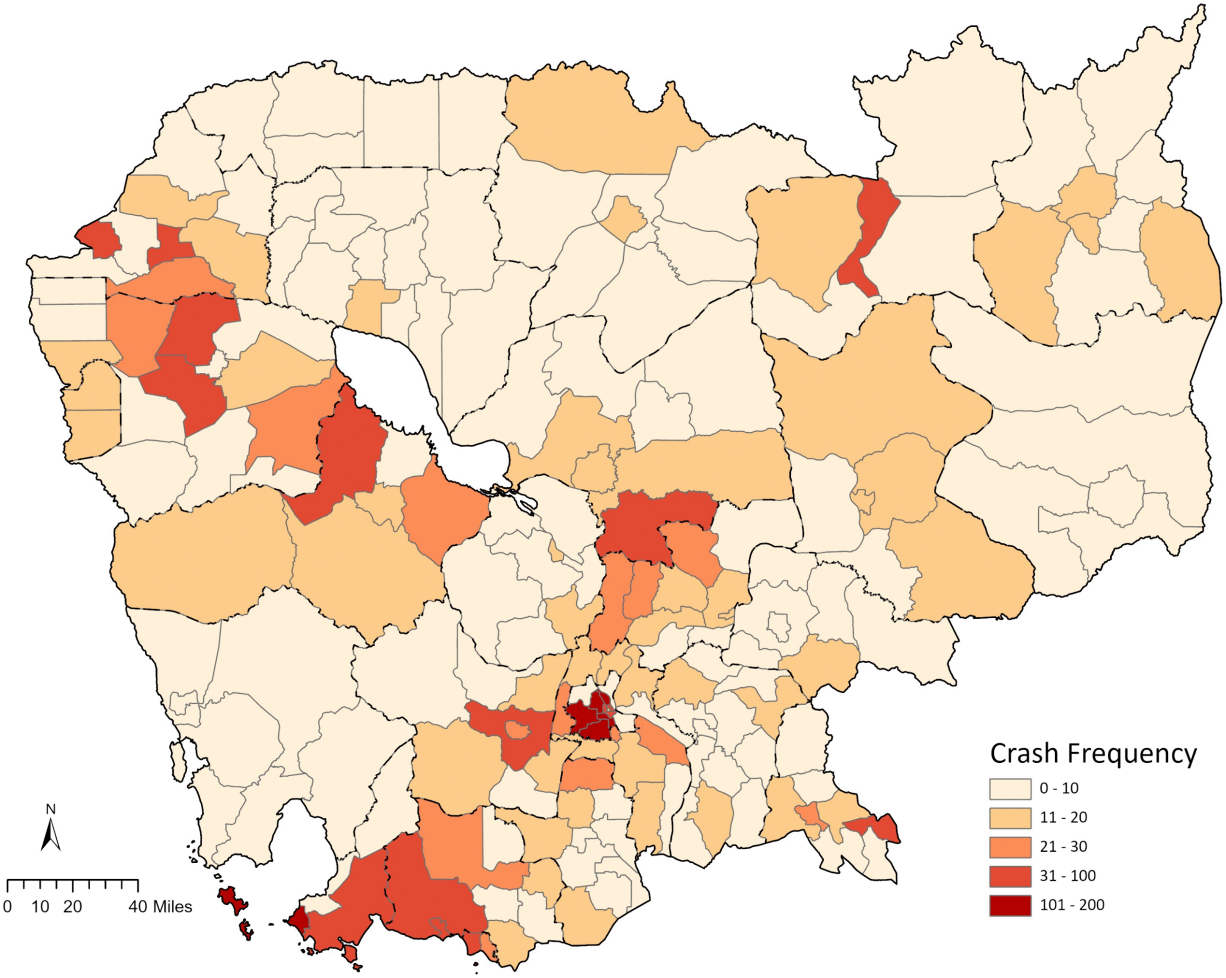
Spatial distribution of motorcycle crash frequency across Cambodia in 2019. (Base map data © OpenStreetMap contributors, licensed under the Open Database License (ODbL); Analytical layers were created by the authors.).

The spatial distribution of motorcycle crash frequency by each district across Cambodia for the year 2019 is shown in Fig 2. This Figure reveals that the central region of Cambodia, which includes parts of Phnom Penh municipality and its surrounding districts, exhibits a notably higher frequency of motorcycle crashes, as well as the southwest coastal area. This is contrasted starkly with the majority of the country’s provinces, which predominantly fall within the lower crash frequency categories of 0–10 and 11–20, indicating a lower occurrence of motorcycle crashes. It is apparent that certain districts, particularly those in the proximity of Phnom Penh, Kandal, and Kampong Thom provinces, are marked by a darker shade. This highlights a moderate to high level of motorcycle crash occurrences, which may warrant further investigation. This spatial distribution further underscores the need for targeted road safety measures and interventions in high-frequency zones, to mitigate the risk of motorcycle crashes.

To further check the spatial autocorrelation of motorcycle crash frequency across Cambodia, its global Moran’s Index (as shown in Equation 1) was calculated using ArcGIS Pro 2.7 software. The derived Moran’s Index suggests a significant clustering pattern within the motorcycle crash frequency, evidenced by a Moran’s I value of 0.447 (as shown in Table 2). Crucially, the statistical significance of this clustering is supported by a p-value of 0.000, which is notably below the 0.01 threshold, and a z-score of 10.302, far exceeding the critical value of 2.58. These results provide a 99% confidence level in rejecting the null hypothesis that the observed spatial distribution is random, thereby indicating a less than 1% probability of a random distribution in the motorcycle crash frequency.

**Fig 3:**
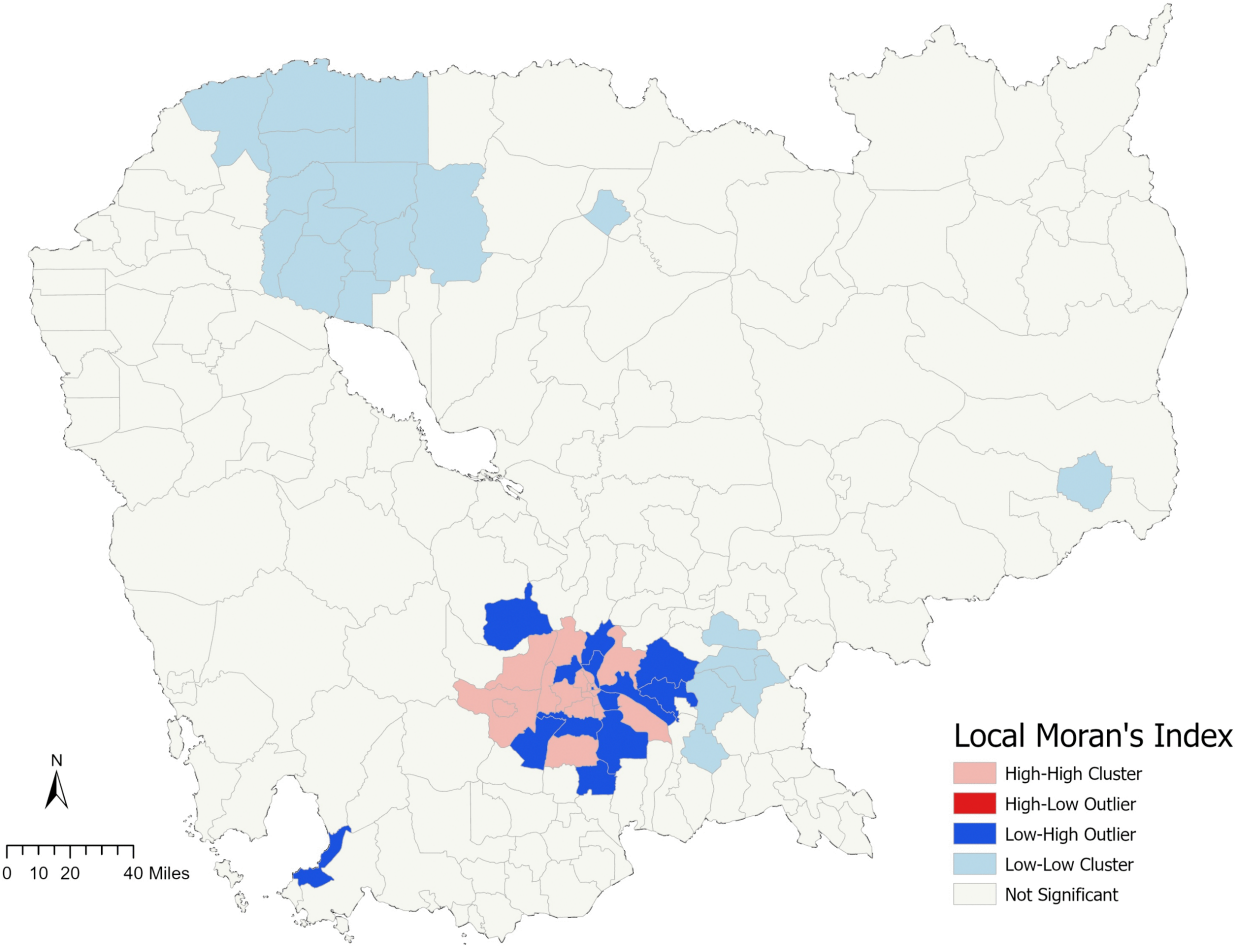
Local indicators of spatial association (LISA) map of motorcycle crash frequency across Cambodia. (Base map data © OpenStreetMap contributors (ODbL); Analytical layers were created by the authors.).

Fig 3 shows the spatial distribution of district-level crash frequency in Cambodia using Local Moran’s Index. This index is a statistical measure of spatial autocorrelation, which means it evaluates whether the pattern expressed is clustered, dispersed, or random across the map. The High-High Cluster (Pink) means regions with high crash frequency surrounded by other areas with high crash frequency. These are clusters where the high crash frequency is similar in a location and its neighbors, representing a significant concentration of crashes. This might be due to factors such as heavy traffic, higher rates of pedestrian and vehicle interaction, or possibly inadequate road safety measures. The Low-Low Cluster (Light Blue) represents regions with low crash frequency surrounded by other areas with low crash frequency, forming a cluster of similar low incidence locations. The light blue areas might correspond to rural or less densely populated regions where the frequency of crashes is relatively low. This could be due to lower traffic volumes, fewer intersections, or potentially less reporting of incidents. The Low-High Outlier (Blue) are areas that have a low crash frequency but are surrounded by areas with high crash frequency. These are considered spatial outliers because their condition is different from their neighbors. The blue areas could be safer zones surrounded by high-risk areas, perhaps due to effective local traffic management, better road conditions, or community-driven safety initiatives. Areas in grey indicate regions, which represents large part of the total 197 districts in Cambodia, the crash frequency does not show significant spatial autocorrelation, meaning that the distribution neither forms clusters nor outliers with statistical significance. The grey areas do not show a clear pattern and might represent regions where the crash frequency is random, or the data is inconclusive. This might be due to inconsistent data collection or diverse conditions that do not lead to a clear spatial correlation. For Cambodia, a developing country with a mix of urban and rural areas, these patterns highlight the necessary of exploring spatial effect of factors on motorcycle crash frequency.

In traditional statistical modeling process, it’s typically assumed that observations are independent and that the relationships between explanatory variables and a response variable remain constant across different spatial locations [5]. However, the reality of crash occurrences contradicts this assumption, as they often exhibit spatial correlation [6–9]. For spatial analyses, crash event data are often aggregated into various levels, from broader regional zones to more specific traffic analysis zones [10], and even down to individual road segments to investigate their spatial correlation with several crash indicators [11]. Zonal crash analyses can provide crucial insights, such as enabling comparative assessments across various zones or pinpointing safety issues within specific areas. Consequently, this information can guide the implementation of targeted safety measures to enhance local traffic safety [12].

Therefore, considering the spatial variation of motorcycle crash frequency, the primary objective of this study is to investigate the spatial pattern of motorcycle crash frequency across Cambodia. More specifically, this study aims to: (a) explore the spatial characteristics and distribution of motorcycle crashes; (b) develop and compare the non-spatial and spatial zonal motorcycle crash frequency regression models; (c) examine the impact of built environments and climate characteristics on motorcycle crash frequency. The findings from this study are intended to not only provide estimates of motorcycle crash frequency by incorporating spatial differences in explanatory variables but also to support efforts in planning, engineering, enforcement, and educational initiatives aimed at improving road safety.

## 2. Literature review

### 2.1. Research on the statistical analysis of crash frequency

Numerous empirical research has evaluated the effect of various attributes on crash frequency in a non-spatial statistical framework, such as Generalized Linear models (GLM), Poisson regression models (PR), Negative Binomial models (NB). These studies have investigated the influence of built environmental and transportation infrastructure on the probability of crash occurrences [13–18], as well as the role of the road characteristics such as arterial roads, rural roads, freeways [19–22], and specific locations like junctions and controlled crossroads [23–25]. Additionally, the impact of climatic attributes including wind strength, visibility, precipitation, light, and annual wet days on crash frequency has also been a subject of research [26–29]. The inclusion of the number of rainy days as a variable has helped in understanding the influence of factors like exposure and pavement condition on crash occurrences [28,30]. However, there has been a lack of comprehensive exploration into spatial variations of these explanatory factors affecting road crash frequency.

Although such non-spatial global regression models are useful to provide information about road crashes, they often assume the independence of variables. Therefore, they fail to adequately address the crucial aspect of spatial autocorrelation, potentially leading to biased results and reduced accuracy when examining the relationship between explanatory and dependent variables. Spatial dependence and spatial heterogeneity are two common spatial elements when considering spatial autocorrelation [6]. Spatial dependence refers to the possibility that the intensity of events at one location may affect the intensity of events at surrounding locations. Spatial heterogeneity means that events may not be evenly distributed across different spaces, such as the incidence of traffic accidents at different locations. These two issues often arise when collecting data by geographical units such as provinces, districts/cities, or villages. Scholars have also shown that the relationship between independent and dependent variables in collision accidents may change with spatial changes. Therefore, analyzing without considering spatial differences may lead to misleading conclusions [31]. Hence, there exists a critical need for further investigation utilizing spatially methodologies to obtain more precise insights into the relationship between the explanatory variables and overall crash occurrences [32].

### 2.2. Research on spatial analysis methodology of crash frequency

Previous studies have introduced various methodologies to assess the spatial impact of explanatory variables on crash frequency. Among these, geographically weighted regression (GWR) stands out as an effective approach, rooted in the fundamental principles of geography [33,34]. GWR enhances analysis by applying weights to nearby observations based on their proximity to a focal point, allowing for the adjustment of regression coefficients to reflect local contexts. This approach enables the exploration of location-specific relationships between dependent and independent variables, offering subtle insights into spatial dependence and heterogeneity. The application of GWR in road safety research has gained considerable recognition in recent years [35,36]. For instance, Mathew et al. [37] demonstrated GWR’s superiority in identifying spatial dependencies and variations in risk factors affecting teen crash frequency, compared to traditional generalized linear models. Similarly, Pirdavani et al. [38] also found that GWR outperforms the OLSR model in spatial analyses, underscoring its value in road safety studies.

Previous research has developed various types of GWR models, including Geographically Weighted Lasso regression (GWLR), Geographically Weighted Poisson regression (GWPR), Geographically Weighted Poisson Quantile regression (GWPQR), and Geographically Weighted Negative Binomial regression (GWNBR) [37,39–41]. These models are particularly useful in handling spatial effects while suitable for various types of data and problems. In dealing with the spatial effect of collision accident data, the GWPR and GWNBR models have shown better results than global non-spatial models [39,42,43]. However, Poisson distribution’s premise is that the mean is equal to the variance, which is not always true for crash data. To address this issue, GWNBR was introduced as a method for modeling counting data with spatial heterogeneity and excessive dispersion [44–45]. It has been widely used in some regional studies to explore the spatial changes of the relationship between explanatory variables and dependent variables [46–48]. In the field of accident analysis, several scholars have carried out research using GWNBR models. Gomes et al. [10] investigated the impact of spatial factors on crash frequency. Their findings indicated that the GWNBR model was better at capturing the spatial heterogeneity of crash frequency than GWPR model. Mathew et al. [37] used GWNBR to study the impact of road environmental factors such as road network, population characteristics and land factors on teen crash frequency. Oluwajana et al. [49] used seven goodness-of-fit indicators to compare the GWPR and GWNBR models, and the results showed that the GWNBR model with fixed bandwidth had the best predictive performance.

Although there are numerous studies on geographic risk factors related to crashes on the regional level, Lee et al. [50] applied Negative Binomial regression models to crash data from the United States and Italy to assess the transferability of models between these two countries. Their findings indicated that models for crashes were not transferable between the two nations, despite sharing several significant variables [51]. Considering this non-transferability between crash frequency analysis in various countries, it is important to explore the motorcycle crash frequency in developing countries like Cambodia.

Overall, research on spatial analysis of motorcycle crash frequency is very limited. Given the challenges in transferring crash frequency analysis findings across different countries, this study seeks to fill the existing research gaps by examining the spatial influence of built environment and climatic factors on motorcycle crash frequency in Cambodia, employing GWR-based methodologies.

## 3. Methodology

In this study, we utilize Poisson and Negative Binomial regression methods to model motorcycle crash frequency due to their proficiency in handling count data. This modelling procedure begins with an assessment of motorcycle crash frequency distribution, emphasizing its mean and variance across the 197 districts of Cambodia. As indicated in Table 1, the average motorcycle crash frequency is 16.152, with a standard deviation of 25.908. Notably, the variance significantly surpasses the mean, leading us to adopt the Negative Binomial distribution for modeling motorcycle crash frequency in Cambodia. Additionally, an enhanced GWNBR model is applied to accurately discern the spatial influences of various explanatory factors on motorcycle crash frequency, thereby offering a more comprehensive understanding of the crash data.

**Table 1:** Descriptive statistics of the variables.

Variables	Description	Min	Max	Mean	S.D.
Motorcycle crash frequency	Motorcycles crash frequency by each district.	0.000	162.000	16.152	25.908
***Exposure***					
Road length (km)	Natural logarithm of the cumulative road length.	1.904	3.191	2.626	0.250
***Built environments***					
Road density (per km^2^)	The density of all types of roads.	0.070	46.369	1.976	4.858
Intersection density (per km^2^)	The density of road intersections.	0.010	424.179	13.748	54.420
Population density (per km^2^)	The population density by each district.	0.756	31978.490	690.457	3180.796
Male population percentage	The percentage of male population.	0.456	0.530	0.488	0.013
Household density (per km^2^)	The household density by each district.	0.156	7009.520	152.672	686.093
Residential area proportion	The proportion of residential area.	0.001	0.958	0.045	0.144
***Climate characteristics***					
Avg_Precipitation (mm/year)	The average precipitation by each district.	1.736	15.339	5.356	2.383
Annual rainy days percentage	The total rainy days proportion by each district.	0.517	0.772	0.609	0.057
Avg_Wind speed (kph/year)	The average wind speed by each district.	5.770	22.968	16.825	4.780

### 3.1. Data preparation

In this section, a spatial motorcycle crash database is compiled with the terms and conditions for the source of the data. The motorcycle crash frequency data, which includes property-damage-only, injury, and fatal crashes, were extracted from Road Crash Victim and Information System (RCVIS) by Cambodian National Road Safety Committee, and the data were analyzed in aggregated, district-level form only. No personal, identifiable, or sensitive information was accessed or processed. For this study, we focused on crashes that occurred in 2019. The frequency of motorcycle crashes within each district was the dependent variable in our analysis. The selection of explanatory variables was guided by prior research and their anticipated influence on motorcycle crashes. This study adopts the “3Ds” framework of built environments – density, diversity, and design [52]. Density encompasses variables like population density, male population proportion, and household density, all of which are commonly used in zonal level traffic safety analyses [37,53–55]. Diversity is represented by land use proportions, residential areas, tourist attractions, green spaces, and cultural and sports facilities, which are identified as significant crash pattern influencers by previous research [56]. The design component includes road network characteristics such as road density, number of intersections, intersection density, and the lengths of major and minor roads, which are key factors affecting crash frequency [57]. Climate and built environment data were obtained from publicly available sources and used in accordance with their respective usage policies.

In this research, the primary spatial unit is the district level, which represents the second-tier administrative division in Cambodia, encompassing a total of 197 districts nationwide. Data regarding the road network, bus stops, administrative boundaries, land use types, and Points of Interest (POI) were sourced from OpenStreetMap (OSM). Using this OSM data, the road length, number of intersections were calculated with ArcGIS software. Population information was obtained from the 2019 General Population Census of the Kingdom of Cambodia, conducted by the National Institute of Statistics under the Ministry of Planning. Furthermore, climate-related data in 2019 were obtained from the VisualCrossing Weather API [58], which provides station-based historical weather observations. To spatially associate climate information with traffic analysis units, climate station data were matched to each district using the nearest-distance principle, whereby each district is assigned the observations from the geographically closest weather station.

Then, the dataset for this study underwent a rigorous data cleaning process for each variable, where observations with missing or omitted values were removed. The final dataset contained 3182 motorcycle crashes in total. The minimum, maximum, mean, and standard deviation of this dependent variable and other 17 explanatory variables are shown in Table 1. The motorcycle crash frequency ranges from 0 to 162 with an average of 16.152 and a standard deviation of 25.908. Demographic data show a male population portion averaging 0.488 with a low standard deviation, suggesting minor variation across districts. Population and household densities vary widely among districts. Built environment characteristics provide details on road network, bus station, residential areas, and amenities like schools and public services, while road network attributes with high stand deviation indicate significant variation among districts. Due to the fact that commercial area and industrial area are not available in many districts in our database, we do not include the commercial area and industrial area in our research. Finally, climate variables like average precipitation, annual rainy days, wind speed, temperature and humidity are reported, both of which could potentially impact crash frequency.

**Fig 4:**
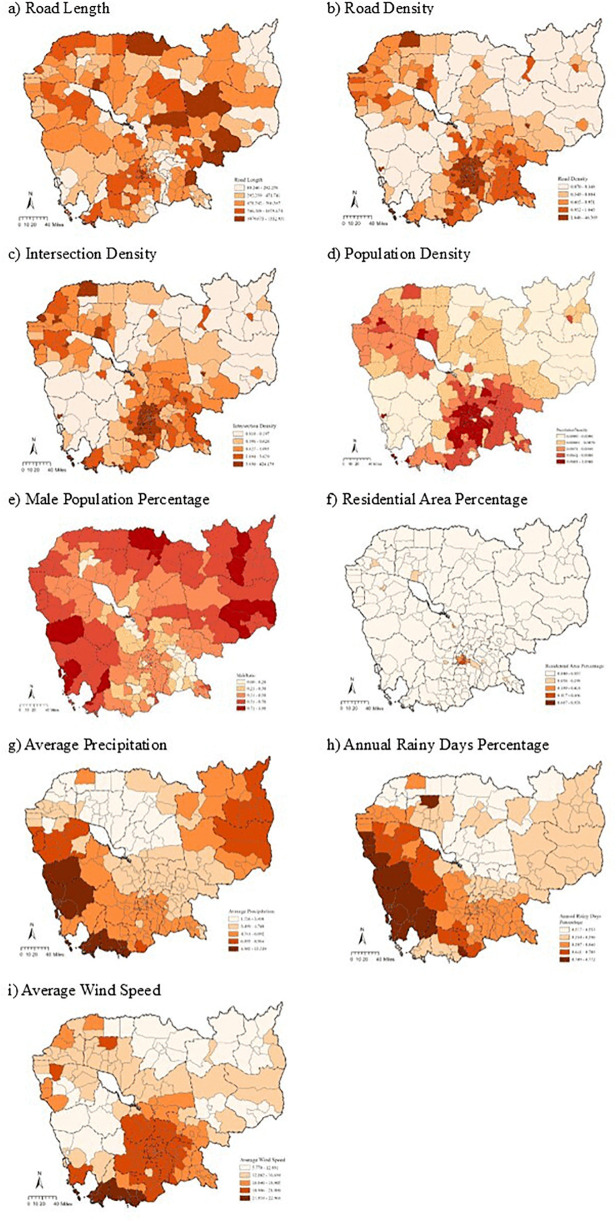
Spatial distribution of built environments and climate characteristics. (Base map data © OpenStreetMap contributors (ODbL); Analytical layers were created by the authors.).

Fig 4 shows the spatial distribution of the built environments and climate characteristics. We could conclude that road density, population density, residential area proportion, and intersection density show large numbers in areas around the capital Phnom Penh, Krong Siem Reap in the northwest area, and the southwest Kampong Som area.

### 3.2. The measurement of spatial dependency - Moran’s Index

In this section, we use Moran’s Index to measure the spatial dependency of each potential explanatory variable. The Moran’s Index is calculated as follows:


$$I=\frac{N\sum_{i=\text{1}}^{N}\;\;\sum_{j=\text{1}}^{N}\;\;w_{ij}\left(x_{i}-\bar{x}\right)\left(x_{j}-\bar{x}\right)}{W\sum_{i=\text{1}}^{N}\;\;{\left(x_{i}-\bar{x}\right)}^{\text{2}}}$$ (1)

where $w_{ij}$ denotes the spatial weight of sample *i* and *j*, representing the spatial proximity or connection between them. *N* denotes the number of spatial units indexed by *i* and *j*. *W* is the sum of all spatial weights $w_{ij}$.

Moran’s Index value is a measure that ranges from −1 to +1, which indicates perfect dispersion and perfect correlation, respectively. A value of 0 suggests that the spatial pattern is random, with no apparent clustering or dispersion. Positive values suggest that similar values occur near each other (spatial autocorrelation), while negative values suggest that dissimilar values occur near each other (spatial dispersion). After checking the value of Moran’s Index, the bus station number, school number, public service number and tourism place number variables were removed from our explanatory dataset due to their non-significant variation among districts. The Moran’s Index test results of the remaining variables are shown in Table 2. The positive z-score values indicate that the data are spatially clustered.

**Table 2:** Moran’s Index and VIF of each variable.

Variables	Moran’s Index	Expected index	Z-score	P-value	VIF
***Crash***					
Motorcycle crash frequency	0.4468	−0.0051	10.3022	0.000	–
***Exposure***					
Road length	0.1501	−0.0051	3.6969	0.000	4.35
***Built environments***					
Road density	0.6053	−0.0051	16.4258	0.000	4.47
Intersection density	0.6299	−0.0051	16.4776	0.000	3.52
Population density	0.4246	−0.0051	12.2852	0.000	1.93
Male population percentage	0.6349	−0.0051	13.9847	0.000	2.00
Household density	0.4188	−0.0051	12.1785	0.000	11.02
Residential area proportion	0.3102	−0.0051	12.9832	0.000	3.98
***Climate characteristics***					
Avg_Precipitation	0.6171	−0.0051	14.5097	0.000	2.22
Annual rainy days percentage	0.6851	−0.0051	15.8521	0.000	1.88
Avg_Wind speed	0.7261	−0.0051	17.4791	0.000	2.04

### 3.3. Geographically weighted negative binomial regression

The Geographically Weighted Negative Binomial Regression (GWNBR) is a statistical model developed by de Silva for analyzing spatial data where the response variable is count-based and potentially over-dispersed (i.e., the variance exceeds the mean) [59]. For the over dispersed data, the GWNBR can model data in a non-stationary manner. The negative binomial distribution has an overdispersion coefficient *α*. The formula for negative binomial regression is as follows:


$$\text{ln}(\mu_{j})=\text{ln}(t_{j})+\sum_{k}\beta_{k}x_{jk}$$ (2)

where $t_{j}$ is an offset variable, $\mu_{j}=E\left(y_{j}\right),\text{ Var}(y_{j})=\mu_{j}+\alpha \overset{\text{2}}{\underset{j}{\mu }}$, *α* is an excessive dispersion coefficient, $\beta_{k}$ is a parameter related to the explanatory variable $x_{k}$, k=1,2⋯K and j=1,2⋯n, $y_{j}$ is the $\;j_{th}$ dependent variable, NB represents negative binomial. This model considers a logarithm link function

GWNBR allows parameters $\beta_{k}$ and *α* to vary with spatial variation. The formula is as follows:


$$y_{j}\sim \text{NB}\left[t_{j}\;\text{exp}\left(\sum_{k}\beta_{k}\left(u_{j},v_{j}\right)x_{jk}\right),\alpha \left(u_{j},v_{j}\right)\right]$$ (3)

where $\left(u_{j},v_{j}\right)$ are the locations (coordinates) of the data points *j*.

Considering the weight of the data near point *i*, the local log-likelihood of GWNBR at *i* has the following formula:


$$\begin{array}{c} L\left(\beta \left(u_{i},v_{i}\right)|x_{jk},y_{j},\alpha_{i}\right)=\sum_{j=\text{1}}^{n}\left\{y_{j}\text{lo}\text{g}\left[\alpha_{i}\mu_{j}\left(\beta \left(u_{i},v_{i}\right)\right)\right]\right]\\ -\left[y_{j}+\text{1}/\alpha_{i}\right]\text{lo}\text{g}\left[\text{1}+\alpha_{i}\mu_{j}\left(\beta \left(u_{i},v_{i}\right)\right)\right]\\ +\text{log }[\Gamma (y_{j}+\text{1}/\alpha_{i})]-\text{log}[\Gamma (\text{1}/\alpha_{i})]-\text{log}[\Gamma (y_{j}+\text{1})]\}w(d_{ij}) \end{array}$$ (4)

where $w(d_{ij})$ is the weight matrix and Γ(x) is an integral term, which can be calculated by the following equation.


$$\begin{array}{c} \Gamma (\text{x})=\int_{\text{0}}^{\infty }\theta^{x-\text{1}}e^{-\theta }d\theta \end{array}$$ (5)

When converging, the following equation can be used to estimate $\beta \left(u_{i},v_{i}\right)$:


$$\begin{array}{c} \hat{\beta }(u_{i},v_{i})\begin{array}{c} \;={\left[{𝐗}^{\prime }𝐖\left(u_{i},v_{i}\right)𝐀\left(u_{i},v_{i}\right)𝐗\right]}^{-\text{1}}{𝐗}^{\prime }\times 𝐖\left(u_{i},v_{i}\right)𝐀\left(u_{i},v_{i}\right)𝐳\left(u_{i},v_{i}\right) \end{array} \end{array}$$ (6)

where $\hat{\beta }(u_{i},v_{i})$ is the coefficient vector estimated from region *i*, 𝐗 is a matrix composed of explanatory variables, $𝐀\left(u_{i},v_{i}\right)$ is the GLM diagonal weighting matrix at convergence, $𝐳\left(u_{i},v_{i}\right)$ is the adjusted dependent variable vector, calculated by the following equation:


$$\begin{array}{c} \begin{array}{c} 𝐳\left(u_{i},v_{i}\right)=X\hat{\beta }\left(u_{i},v_{i}\right)+\frac{y_{j}-\mu_{j}\left(\hat{\beta }\left(u_{i},v_{i}\right)\right)}{a_{ij}\left(\text{1}+\alpha_{i}\times \mu_{j}\left(\hat{\beta }\left(u_{i},v_{i}\right)\right)\right)} \end{array} \end{array}$$ (7)

The best fit of the model was assessed using Akaike Information Criterion (AIC). The lower value of AIC indicates a better fit of the model [60].

Another important parameter in evaluating the GWNBR model is the bandwidth. The selection of bandwidth and corresponding kernel function will affect the performance of the model. The rate at which bandwidth control data point weights decrease with increasing distance regression points. When the bandwidth is large, the weight slowly decays, and vice versa. The AIC and Cross-validation (CV) are two common evaluation indicators for finding the optimal bandwidth. The optimum AIC aims to identify the most suitable model by selecting a bandwidth that minimizes both the discrepancies between observed and predicted values and the complexity of the model [61]. Concurrently, the optimal CV method aims to pinpoint the best model by choosing a bandwidth that reduces the divergence between observed and fitted values through the cross-validation technique [61]. Therefore, when searching for the optimal bandwidth, it is necessary to reference the values of both indicators.

### 3.4. Measurement of goodness of fit

The three commonly used indicators for evaluating and comparing the performance of GWNBR models are AIC, Mean Absolute Deviation (MAD), and Root Mean Squared Error (RMSE). The AIC, MAD and RMSE indicators are defined as follows:


$$\text{AIC}=-\text{2ln}(\hat{L})+\text{2}k$$ (8)

where $\hat{L}$ is the maximum estimate of log likelihood and *k* is the degree of freedom.


$$\begin{array}{c} MAD=\frac{\sum_{i=\text{1}}^{n}\left|\hat{y_{j}}-y_{\text{j }}\right|}{n} \end{array}$$ (9)

$$\begin{array}{c} RMSE=\sqrt{\frac{\sum_{i=\text{1}}^{n}{\left({\hat{y}}_{\text{j }}-y_{j}\right)}^{\text{2}}}{n}} \end{array}$$ (10)

where *n* is the number of observations, $y_{j}$ is the observed number of crashes, $\begin{array}{c} {\hat{y}}_{\text{j}} \end{array}$ is the predicted number of crashes. The lower the values of AIC, MAD, and RMSE, the better the model performance.

## 4. Results and discussion

To further enhance the reliability of our models, assessing the multi-collinearity among independent variables was deemed crucial. High correlation among independent variables in a regression model can lead to skewed results and reduce the model’s reliability [62]. In this study, we employed the Variance Inflation Factor (VIF) to test multi-collinearity. VIF values, which are indicative of the level of collinearity, suggest that higher values correspond to stronger collinearity [63,64]. A VIF range between 0–10 is generally considered indicative of low collinearity. The multi-collinearity test results for the initially chosen 16 explanatory variables are presented in Table 2. Due to their VIF values exceeding 10, “Household density” and “Health service number” were excluded from the preliminary explanatory variables [64].

### 4.1. The overall results of the global model and GWNBR models

In our research, non-spatial Ordinary Least Squares regression (OLSR), Poisson regression (PR), and Negative Binomial Regression (NBR) models were established as foundational comparisons for GWNBR models. Key variables identified in the NBR model informed the development of GWNBR models. These GWNBR models were particularly effective in assessing how various factors differently influence motorcycle crash frequency across Cambodia’s districts, highlighting the spatial variability of the relationship between the dependent variable and key explanatory variables.

Selecting the appropriate bandwidth and kernel function is pivotal in the calibration of GWNBR models [38]. GWNBR offers two kernel options: fixed or adaptive. With a fixed kernel, neighboring data points are selected based on a predefined distance criterion, such as including all neighbors within a 50 km radius. Conversely, the adaptive kernel’s selection of neighbors is guided by optimal values determined by the AIC or CV [61]. In this study, we opted for the adaptive kernel due to its flexibility in adjusting the spatial extent to encompass a broader geographic area, ensuring a consistent number of observations within the kernel’s scope.

The SAS/IML© macros [65] were employed in our study to develop the GWNBR models. The GWNBR models require the determination of the optimum bandwidth for model calibration. Thus, in this study, we determined the optimum bandwidth of GWNBR by adopting both the optimum AIC and CV indicators. The golden section search method was employed to find the optimal bandwidth in our study.

Table 3 shows the performance metrics for both non-spatial and spatial models, focusing on Likelihood, AIC, RMSE, and MAD. The results highlight the GWNBR model with adaptive bandwidth and optimized AIC as superior compared to other models, as evidenced by its lower MAD, RMSE, and AIC values. This confirms the necessity of considering the spatial variation and over dispersion that exists in the motorcycle crash data. The MAD values increase from 3.031 in the GWNBR model with adaptive optimized CV indicator to 4.224 in the NBR model, which further marks a notable enhancement in the model’s ability to account for variability. Additionally, the AIC values for the GWNBR model with adaptive AIC are markedly lower compared to those of the global models, highlighting the superior efficiency of the GWNBR approach. This suggests that incorporating spatial variation into the analysis can notably enhance the explanatory power of the model. The detailed outcomes derived from the GWNBR model with adaptive AIC will be thoroughly examined in the subsequent section.

**Table 3:** Performance comparison of each model.

Models	Type	Bandwidth	Likelihood	AIC	RMSE	MAD
OLSR	–	–	−1228.129	2485.045	23.214	12.192
PR	–	–	−817.091	1662.715	17.436	9.197
NBR	–	–	−694.513	1394.088	14.872	8.827
GWNBR1	Adaptive-AIC	175	−413.123	965.323	7.795	5.450
GWNBR2	Adaptive-CV	186	−682.472	1313.331	12.819	8.524

Diagnostic statistics further justify the progressive transition from PR to NBR framework. For the PR model, the dispersion statistic (χ²/df = 12.3) substantially exceeds the conventional threshold of unity, indicating severe overdispersion and rendering the Poisson assumption inappropriate for the motorcycle crash data. To address this issue, a NBR model was estimated; however, Moran’s I test on the global NBR residuals showed significant positive spatial autocorrelation (Moran’s I = 0.5007, z = 12.0523, p < 0.01), suggesting that spatial heterogeneity remained inadequately captured. In contrast, the GWNBR model with an adaptive bandwidth selected by the AIC criterion effectively mitigated residual spatial dependence, with Moran’s I reduced to 0.0301 and no longer statistically significant (p = 0.265). This indicates that allowing regression parameters to vary spatially substantially improves model adequacy. Moreover, relative to the global NBR model, the GWNBR model achieved a 44.2% reduction in RMSE (from 14.872 to 7.795) and a marked decrease in AIC (from 1394.088 to 965.323). These results demonstrate that the incorporation of both overdispersion and spatial non-stationarity yields improvements that are not only statistically significant but also practically meaningful, thereby providing strong empirical support for the adoption of the GWNBR framework.

### 4.2. Local estimates

**Fig 5:**
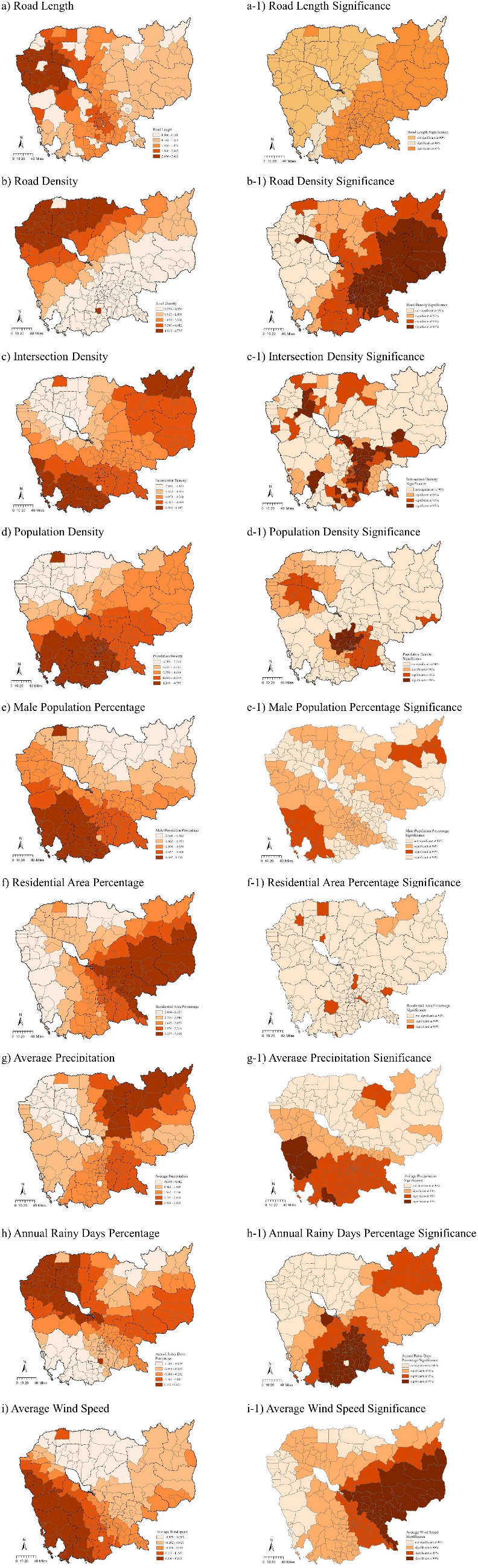
Local estimates of built environments and climate from GWNBR model. (Base map data © OpenStreetMap contributors (ODbL); Analytical layers were created by the authors.).

This section explores the spatial association variations between various built environments, climate characteristics variables and motorcycle crash frequency are analyzed and presented in Table 4 and Fig 5. Only the local estimates of each explanatory variable by the best GWNBR with adaptive bandwidth and optimal AIC model will be presented. Five statistical measures are utilized to show the non-stationary spatial effect of each explanatory variable: the minimum value (MIN), 25th percentile lower quartile (LQ), median, 75th percentile upper quartile (UQ), and maximum value (MAX), as detailed in Table 4. The local coefficients across various districts exhibit diverse and sometimes surprising directional tendencies, demonstrating the GWNBR model’s capability at capturing spatial non-stationarity. In some districts, a particular independent variable may show a statistically significant relationship with motorcycle crash frequency, whereas in others, the relationship may not be significant. Additionally, the LQ, Median, and UQ values reveal that some built environment and climate characteristic estimates fluctuate from negative to positive, a complexity not observable in non-spatial models such as the OLSR, PR, and NBR models. This phenomenon is consistently observed in the outcomes of the GWNBR models, where both positive and negative coefficients were estimated across all zones, indicating the complex and variable nature of the spatial relationships being analyzed.

**Table 4:** Estimation results of the GWNBR model for local variables.

Variable	Minimum	LQ	Median	UQ	Maximum
Intercept	0.848	1.337	1.406	1.666	2.017
***Exposure***					
Road length	0.118	1.231	1.700	2.215	3.461
***Built environments***					
Road density	0.356	0.548	0.688	3.710	6.820
Intersection density	−2.035	−0.996	−0.251	−0.104	0.398
Population density	−4.294	−2.333	−1.278	−1.043	−0.705
Male population percentage	−2.275	−1.119	−0.569	−0.380	0.142
Residential area proportion	2.086	2.514	3.006	3.286	3.482
***Climate characteristics***					
Avg_Precipitation	0.171	1.344	2.031	2.379	3.946
Annual rainy days percentage	−1.285	−0.720	−0.452	−0.029	0.707
Avg_Wind speed	−0.551	−0.071	0.065	0.216	0.541

#### 4.2.1. Built environments.

Table 4 and Fig 5 present the spatial distribution of local GWNBR parameter estimates and their statistical significance for built environment variables. By examining the proportion of districts with positive versus negative coefficients, this study assesses the degree of spatial non-stationarity across explanatory variables. When a high proportion of districts (approximately 70% or more) exhibit coefficients with the same sign, this suggests a relatively homogeneous association pattern between the built environment factor and motorcycle crash frequency, although the strength and significance of these associations may still vary spatially. Based on the GWNBR results, seven built environment variables demonstrate statistically significant associations with motorcycle crash frequency in at least part of the study area, and these patterns are discussed below with explicit reference to statistical significance.

With respect to the exposure variable, road length, exhibits strong positive associations with motorcycle crash frequency, and statistically significant associations are observed primarily in southeast districts (Fig 5a). Roads often coincide with economic corridors or intercity connections, and districts with higher major road length tend to exhibit stronger associations with motorcycle crash frequency in these regions. In contrast, several districts with extensive road infrastructure show weak or insignificant associations, suggesting that factors such as enforcement intensity, road design standards, or safety interventions may moderate this relationship. These findings underscore the spatially heterogeneous nature of the association rather than implying a uniform effect of major roads on crash occurrence.

Road density shows predominantly positive coefficients across most districts, with a large proportion reaching statistical significance (Fig 5b). This spatial pattern aligns with previous findings [10], indicating that districts with denser road networks tend to exhibit higher motorcycle crash frequencies. In the current analysis, higher road density is associated with increased crash frequency, particularly in and around Phnom Penh and other major urban areas. While denser networks may coincide with more complex traffic environments and higher exposure levels, this study does not infer causality, and the observed associations likely reflect a combination of network structure, traffic volume, and urban activity intensity.

Intersection density exhibits both positive and negative local coefficients, with statistically significant associations observed in spatially clustered districts. Although higher intersection density is often expected to be associated with increased crash occurrence, the predominantly negative associations identified in this study—particularly in districts where the estimates are statistically significant—suggest a more nuanced relationship in the Cambodian context. The lower and upper quartile values indicate that negative associations dominate overall, consistent with previous research [57,66–69]. One plausible interpretation is that higher intersection density reflects a finer-grained street network characterized by shorter block lengths, lower operating speeds, and more frequent points of conflict awareness. In such environments, riders may adopt more cautious behavior due to frequent stopping, increased visual scanning, and the presence of traffic control devices, which may be associated with lower motorcycle crash frequencies. This interpretation is consistent with urban planning literature that highlights the role of dense intersection networks in traffic calming and speed moderation, particularly in mixed-traffic environments [70–72]. Conversely, positive associations observed in a limited number of districts, which are consistent with findings reported by Barua et al. [73] and Jeon et al. [74], indicating that the relationship between intersection density and motorcycle crashes is spatially non-stationary and context-dependent. Differences in intersection design quality, enforcement levels, and rider compliance may partially explain these variations. Overall, the results suggest that intersection density does not exert a uniform influence on motorcycle crash frequency, and its association appears to be shaped by localized network and behavioral conditions rather than a single dominant mechanism.

Population density exhibits consistently negative coefficients across all 197 districts, with statistically significant effects concentrated mainly in the northwestern regions. This spatial pattern is broadly consistent with some previous studies [32,75], although it differs from others [14,17]. The magnitude of the negative association gradually weakens moving outward from the northwest, indicating spatial heterogeneity in the relationship. Importantly, interpretation is limited to districts where coefficients are statistically significant. In these areas, higher population density is associated with lower motorcycle crash frequency. One possible interpretation—offered cautiously—is that higher-density districts may feature urban environments with greater reliance on walking or four-wheel vehicles rather than motorcycles, as well as lower average travel speeds due to congestion. Such contextual factors may contribute to the observed association but cannot be confirmed within the scope of this cross-sectional analysis. Future research incorporating additional indicators, such as sidewalk length or modal share, would help further clarify these relationships.

The male population percentage is associated with predominantly negative coefficients in most districts; however, statistically significant effects are observed in only three districts nationwide. Given this limited spatial significance, interpretations are necessarily restrained. The spatial comparison of Figs 4 and 5e suggests that districts with higher male population shares often coincide with lower motorcycle crash frequencies, particularly in peripheral regions. This pattern may reflect differences in regional socioeconomic structure, travel behavior, or motorcycle usage rates, but these explanations remain speculative. Overall, the limited statistical significance indicates that male population percentage plays a relatively minor and spatially localized role in explaining motorcycle crash frequency.

Regarding residential land use, Table 4 and Fig 5f indicate positive coefficients in several districts, with statistically significant associations observed primarily in eastern and central areas. These results are consistent with prior studies reporting positive associations between residential land use intensity and traffic crashes [76,77], as well as between household concentration and motorcycle crashes [37]. In the present study, districts with a higher proportion of residential land use are associated with higher motorcycle crash frequencies in areas where coefficients are statistically significant. The spatial variation in coefficient magnitude suggests that this relationship is not uniform across Cambodia. For example, strong associations in certain northwest districts may reflect local characteristics such as concentrated residential development combined with limited public service infrastructure. However, these interpretations should be viewed as contextual explanations rather than causal conclusions.

#### 4.2.2. Climate characteristics.

In this research, due to the climatic data availability and multicollinearity between explanatory variables, we only focus on the average precipitation, annual rainy days percentage, and average wind speed variables.

Average precipitation shows a clear south-to-north spatial gradient (Fig 5g), consistent with regional climatic patterns. Statistically significant positive associations with motorcycle crash frequency are concentrated in northern districts. In these areas, higher precipitation levels are associated with higher crash frequencies, in line with prior research identifying rainfall as a correlate of crash occurrence [78–80]. However, in districts where coefficients are not statistically significant, no substantive interpretation is offered. The spatial heterogeneity suggests that precipitation-related effects on motorcycle crashes vary with local travel behavior and infrastructure conditions.

Annual rainy days percentage exhibits predominantly negative associations with motorcycle crash frequency across much of Cambodia (Fig 5h), although positive and statistically significant associations emerge in several districts, particularly in the northwest. In districts with significant negative coefficients, a greater number of rainy days is associated with lower crash frequency, potentially reflecting reduced motorcycle usage or more cautious riding behavior during frequent rainfall. Conversely, in districts with significant positive associations—consistent with previous studies [28,30,81]—terrain characteristics and prolonged exposure to wet conditions may play a role. These mixed patterns highlight the importance of spatial context in understanding weather–crash relationships.

However, the contrasting associations observed for annual rainy days percentage and average precipitation highlight an important behavioral dimension in motorcycle crash occurrence. While precipitation intensity shows a positive association with motorcycle crash frequency in several districts, the number of annual rainy days is predominantly negatively associated with crashes in many areas where the coefficients are statistically significant. This contrast suggests that these two climate indicators may capture different underlying processes. A higher frequency of rainy days may be associated with adaptive or preventive travel behavior, such as reduced motorcycle usage [82,83], increased caution, or lower riding speeds over prolonged periods of exposure to wet conditions. In contrast, precipitation intensity represents an immediate physical hazard, potentially affecting road surface friction and visibility, which may be associated with increased crash risk during rainfall events. The coexistence of negative associations for rainy day frequency and positive associations for precipitation intensity underscores the importance of distinguishing between behavioral adaptation and physical exposure when interpreting climate–crash relationships. These findings indicate that riders may adjust their behavior in response to frequent adverse weather, whereas intense rainfall events pose short-term risks that are less amenable to behavioral compensation. The spatial variability of these associations further suggests that local environmental and infrastructural conditions mediate how climate factors relate to motorcycle crash frequency.

Average wind speed demonstrates both positive and negative associations with motorcycle crash frequency across districts (Fig 4i). Significant negative associations are mainly observed in northern and northeastern regions, while significant positive associations are concentrated in southwestern districts. These contrasting patterns suggest that wind-related effects on motorcycle crashes are highly context-dependent and may be influenced by local topography, urban form, and exposure conditions. Similar associations between wind conditions and crash occurrence have been reported by Hermans et al. [81], although causal mechanisms remain uncertain.

Finally, the spatially varying intercepts in the GWNBR model may capture the influence of unobserved factors not explicitly included in the analysis, such as income level [38], unemployment [84], education [85], or traffic volume [86,87]. The relatively small adaptive bandwidths further indicate strong local sensitivity of motorcycle crash occurrence to spatial context [56].

## 5. Conclusions and policy recommendations

This study examined the spatially varying associations between built environment characteristics, climatic conditions, and motorcycle crash frequency across 197 districts in Cambodia. By comparing global models (OLS, Poisson regression, and negative binomial regression) with a geographically weighted negative binomial regression (GWNBR) model, the analysis demonstrates that motorcycle crash–environment relationships are characterized by pronounced spatial non-stationarity. The superior performance of the GWNBR model highlights its suitability for modeling overdispersed crash count data with spatially heterogeneous effects, offering a more nuanced understanding of local safety patterns than conventional non-spatial approaches.

The results indicate that the direction, magnitude, and statistical significance of associations between explanatory variables and motorcycle crash frequency vary considerably across districts. While many built environment and climate variables exhibit locally differentiated associations, several factors display relatively consistent patterns in a large proportion of districts. Higher road density, longer major and minor road networks, greater residential land-use intensity, and higher precipitation levels are generally associated with higher motorcycle crash frequencies in districts where effects are statistically significant. In contrast, higher population density, greater intersection density, and a higher number of annual rainy days tend to be associated with lower crash frequencies in many districts. These findings reinforce the importance of accounting for spatial heterogeneity when analyzing motorcycle safety outcomes, particularly in low- and middle-income country contexts.

From a policy perspective, the findings suggest that motorcycle safety interventions in Cambodia should move beyond uniform, nationwide strategies and instead adopt a spatially differentiated approach. Districts characterized by dense road networks and extensive road infrastructure may benefit from targeted enforcement, speed management measures, and context-sensitive road design interventions. In areas where residential land use is strongly associated with higher crash frequency, integrating road safety considerations into land-use planning—such as traffic calming in residential zones and improved access management—may be particularly relevant. Conversely, districts where higher intersection density is associated with lower crash frequency may reflect the presence of traffic-calming effects or increased rider vigilance, suggesting that intersection design and control strategies could play a protective role in certain contexts. Climate-related findings further indicate that safety strategies should distinguish between long-term exposure to frequent rainfall, which may be associated with adaptive riding behavior, and high-intensity precipitation events that pose immediate physical hazards. Weather-responsive enforcement, public awareness campaigns, and infrastructure measures such as improved drainage and surface maintenance could therefore be tailored to local climatic conditions.

Several limitations should be acknowledged when interpreting these findings. First, the analysis relies on district-level zonal data, which may give rise to ecological fallacy, whereby associations observed at the aggregate level do not necessarily reflect individual-level risk relationships [88]. Second, although the crash data are aggregated and comprehensive at the district scale, under-reporting—particularly for property-damage-only motorcycle crashes—may affect the observed crash frequencies and introduce bias. Third, as with all geographically weighted models, the GWNBR framework identifies spatially varying associations rather than causal effects; the results should therefore be interpreted as indicative of spatial patterns rather than definitive causal mechanisms. Fourth, the analysis focuses on variables exhibiting spatial variation across districts; future work could explore semi-parametric GWNBR specifications to jointly model spatially varying and spatially invariant factors. Fifth, the absence of detailed traffic exposure data, such as average annual daily traffic (AADT) [28,37,89], limits the ability to directly control for traffic volume effects. Improved availability of exposure and land-use data—including enhanced OpenStreetMap coverage in Cambodia—would further strengthen future analyses.

Despite these limitations, this study demonstrates the value of spatially explicit modeling for understanding motorcycle crash patterns in Cambodia. By highlighting localized associations between the built environment, climate, and motorcycle crashes, the findings provide a foundation for more targeted, context-sensitive safety planning and offer methodological insights applicable to other low- and middle-income countries facing similar road safety challenges.
